# Targeting CREB-binding protein overrides LPS induced radioresistance in non-small cell lung cancer cell lines

**DOI:** 10.18632/oncotarget.25665

**Published:** 2018-06-22

**Authors:** Mira Y. Gökyildirim, Ulrich Grandel, Katja Hattar, Gabriele Dahlem, Elena Schuetz, Florian H. Leinberger, Fabian Eberle, Ulf Sibelius, Friedrich Grimminger, Werner Seeger, Rita Engenhart-Cabillic, Ekkehard Dikomey, Florentine S.B. Subtil

**Affiliations:** ^1^ Department of Internal Medicine IV/V, Universities of Giessen and Marburg Lung Center, Member of the German Center for Lung Research, Giessen, Germany; ^2^ Department of Radiotherapy and Radiooncology, Philipps-University, Marburg, Germany; ^3^ Department of Internal Medicine II, Universities of Giessen and Marburg Lung Center, Member of the German Center for Lung Research, Giessen, Germany; ^4^ Department of Radiotherapy, Universities of Giessen and Marburg Lung Center, Member of the German Center for Lung Research, Giessen, Germany; ^5^ Asklepios Klinik Lich, Lich, Germany; ^6^ Max-Planck Institute for Heart and Lung Research, Bad Nauheim, Germany

**Keywords:** lung cancer, X-irradiation, infection, LPS, CREB

## Abstract

Non-small cell lung cancer (NSCLC) has a very poor prognosis even when treated with the best therapies available today often including radiation. NSCLC is frequently complicated by pulmonary infections which appear to impair prognosis as well as therapy, whereby the underlying mechanisms are still not known. It was investigated here, whether the bacterial lipopolysaccharides (LPS) might alter the tumor cell radiosensitivity. LPS were found to induce a radioresistance but solely in cells with an active TLR-4 pathway. Proteome profiling array revealed that LPS combined with irradiation resulted in a strong phosphorylation of cAMP response element-binding protein (CREB). Inhibition of CREB binding protein (CBP) by the specific inhibitor ICG-001 not only abrogated the LPS-induced radioresistance but even led to an increase in radiosensitivity. The sensitization caused by ICG-001 could be attributed to a reduction of DNA double-strand break (DSB) repair.

It is shown that in NSCLC cells LPS leads to a CREB dependent radioresistance which is, however, reversible through CBP inhibition by the specific inhibitor ICG-001. These findings indicate that the combined treatment with radiation and CBP inhibition may improve survival of NSCLC patients suffering from pulmonary infections.

## INTRODUCTION

Lung cancer is the leading cause of cancer-related death in both men and women in the western hemisphere [1]. With a fraction of 80 to 85% non-small cell lung cancer (NSCLC) is the most prevalent type of lung cancer [2].

NSCLC is generally treated by a complex combination of surgery, radiotherapy and chemotherapy. But, despite the tremendous progress made in all three disciplines the overall survival of NSCLC patients is still very low with a five-year survival rate below 20% [3]. The outcome of these patients is getting even from bad to worse, when NSCLC is associated with a pulmonary infection, which is unfortunately the case in about 70% of lung cancer patients [4]. When no pulmonary infections were present the overall survival of lung cancer patients as determined 28 months after treatment was still above 30%, in contrast to only 10% when patients were suffering from infections [5].

The most common pathogens found in patients with lung cancer are gram-negative bacteria such as *Haemophilus influenza* and *Escherichia coli* [4, 6]. Lipopolysaccharides (LPS), the so called “endotoxin” of gram-negative bacteria components of cell wall are the major bacterial pathogenicity factors [7, 8].

It was previously shown by us [9] that LPS are able to stimulate tumor growth both *in vitro* as well as *in vivo*. Such a pro-proliferative effect of LPS was also reported for lung, liver, ovarian, gastric as well as breast cancer [9–14]. These data might in part explain the negative impact of pulmonary infections on the outcome of NSCLC patients. However, LPS may also worsen the prognosis of these patients via a reduction of the treatment response to radiation or chemotherapy. But so far data are lacking on any interaction between LPS and radio- or chemoresistance.

Cancer cells respond to irradiation in a heterogeneous manner depending on their intrinsic properties (DNA repair capability) or extrinsic environment (hypoxia, e.g.) [15, 16]. The main irradiation induced damage are DNA double strand breaks (DNA-DSBs), which when either mis- or non-repaired may result in a decreased clonogenicity of tumor cells and thereby affecting tumor cell survival [16–18].

It is well known, that after ligation of LPS to the CD14 molecule [19], cellular activation is initiated by binding of this complex to the toll-like receptor 4 (TLR-4) [20–23]. Through this complex LPS are able to activate various MAPK pathways such as ERK, JNK, p38 and the IKK pathway. The MAPK pathways directly or indirectly phosphorylate and activate various transcription factors, including Elk-1, c-Jun, c-Fos, ATF-1, ATF-2, SRF, and CREB. Some of these pathways are already known to have a strong effect on cell proliferation, survival, inflammation, immune regulation as well as DNA repair also including repair of DNA-DSBs [24, 25]. The LPS induced pathways via TLR-4 were also found strongly to depend on a crosstalk to EGFR [26], which is clearly known to be involved in DSB repair [27, 28]. These data suggest that LPS may affect DSB repair either via one of these transcription factors mentioned above or indirectly via EGFR.

Therefore we tested the hypothesis, in how far LPS may affect the cellular response to radiotherapy by reducing its effect on cell survival due to a depressed repair of DNA-DSBs.

## RESULTS

### No effect of LPS on colony forming ability

**Figure 1:**
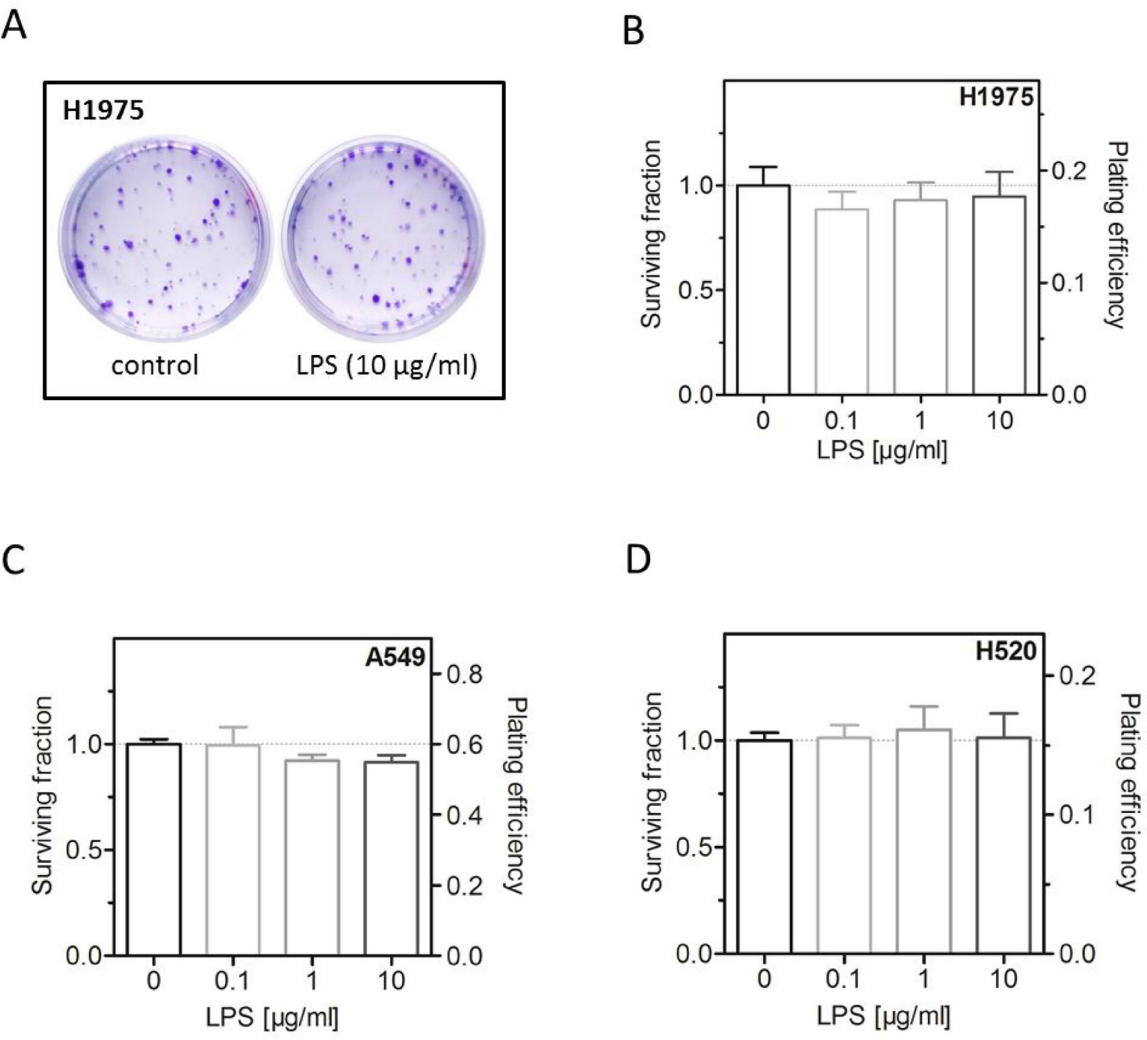
LPS have no effect on colony forming ability (**A**) Representative images of colonies formed of LPS- (10 μg/ml) or sham-treated (control) H1975 cells. (**B**) Survival fraction and plating efficiency of LPS- (0.1, 1, 10 μg/ml) or sham-treated (control) H1975 cells. (**C**) Survival fraction and plating efficiency of LPS- (0.1, 1, 10 μg/ml) or sham-treated (control) A549 cells. (**D**) Survival fraction and plating efficiency of LPS- (0.1, 1, 10 μg/ml) or sham-treated (control) H520 cells. Data are presented by mean ± SEM, *n* ≥ 3.

The effect of the LPS induced pathway via TLR-4 is known strongly to depend on a crosstalk to EGFR [26]. Therefore, this study was performed with three cell lines clearly differing in these parameters. Two cell lines (A549, H1975) with high expression of TLR-4 were selected with the first being EGFR wild-type and the second carrying an EGFR driver mutation; while the third cell line (H520) shows a very low expression of TLR-4 and is EGFR-deficient (Table 1). In a previous work of our group, it was shown that LPS are able to stimulate proliferation of A549 cells [9]. It is now shown here that this treatment does, however, not result in an increased plating efficiency and respectively colony forming ability, neither for A549 nor the other two cell lines (Figure 1).

**Table 1:** Basal expression of *TLR-4-* and *EGFR*-mRNA in H1975, A549 and H520 cells

	H1975 (–ΔCt value)	A549 (–ΔCt value)	H520 (–ΔCt value)
*TLR-4* mRNA	–7.69 ± 0.23	–7.25 ± 0.25	–10.20 ± 0.57
*EGFR* mRNA	2.63 ± 0.20	0.60 ± 0.12	–8.20 ± 0.58

Data are presented as mean ± SEM (*n* = 3).

### LPS induce radioresistance in H1975 and A549, but not in H520 cells

**Figure 2:**
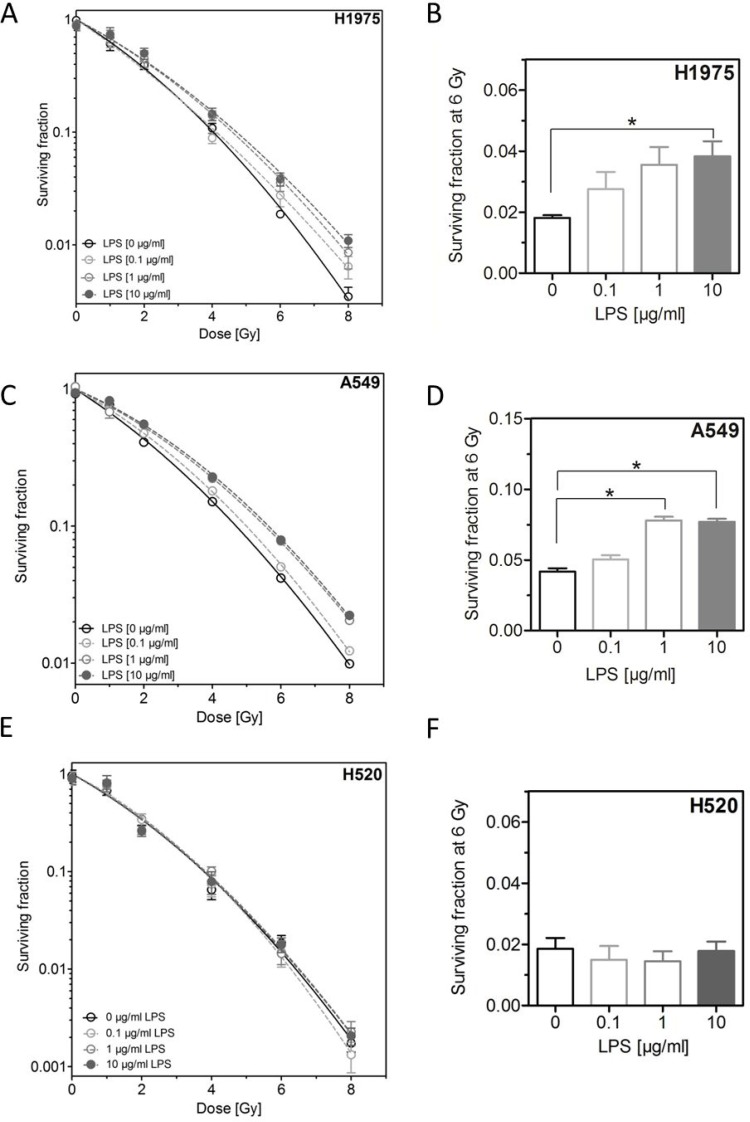
LPS induce radioresistance in H1975 and A549, but not in H520 cells (**A**) Survival fractions of LPS- (0.1, 1, 10 μg/ml) or sham-treated (control) H1975 cells after irradiation with 0–8 Gy. (**B**) Survival fractions at 6 Gy of LPS- (0.1, 1, 10 μg/ml) or sham-treated (control) H1975 cells. (**C**) Survival fractions of LPS- (0.1, 1, 10 μg/ml) or sham-treated (control) A549 cells after irradiation with 0–8 Gy. (**D**) Survival fractions at 6 Gy of LPS- (0.1, 1, 10 μg/ml) or sham-treated (control) A549 cells. (**E**) Survival fractions of LPS- (0.1, 1, 10 μg/ml) or sham-treated (control) H520 cells after irradiation with 0–8 Gy. (**F**) Survival fractions at 6 Gy of LPS- (0.1, 1, 10 μg/ml) or sham-treated (control) H520 cells. Data are presented by mean ± SEM, *n* ≥ 3; ^*^p < 0.05 for comparison versus control, as determined by ANOVA following by Bonferroni's Multiple Comparison Test.

Next, we investigated the effect of LPS on cellular radiosensitivity. Cells were incubated with different concentrations of LPS for 16 h before exposed to X-ray doses up to 8 Gy followed by further incubation for colony growth. Interestingly, LPS were found to induce a radioresistance in H1975 and A549 cells but not in H520 cells (Figure 2E and 2F). For the first two cell lines this radioresistance was already apparent at low doses and clearly increased for higher radiation doses (Figure 2A and 2C). For H1975 and A549 cells a significant increase in radioresistance was obtained at 6 Gy for the concentration of 10 μg/ml LPS, respectively (Figure 2B and 2D).

### Strong up-regulation of CREB dependent pathway after combined treatment with LPS and irradiation

**Figure 3:**
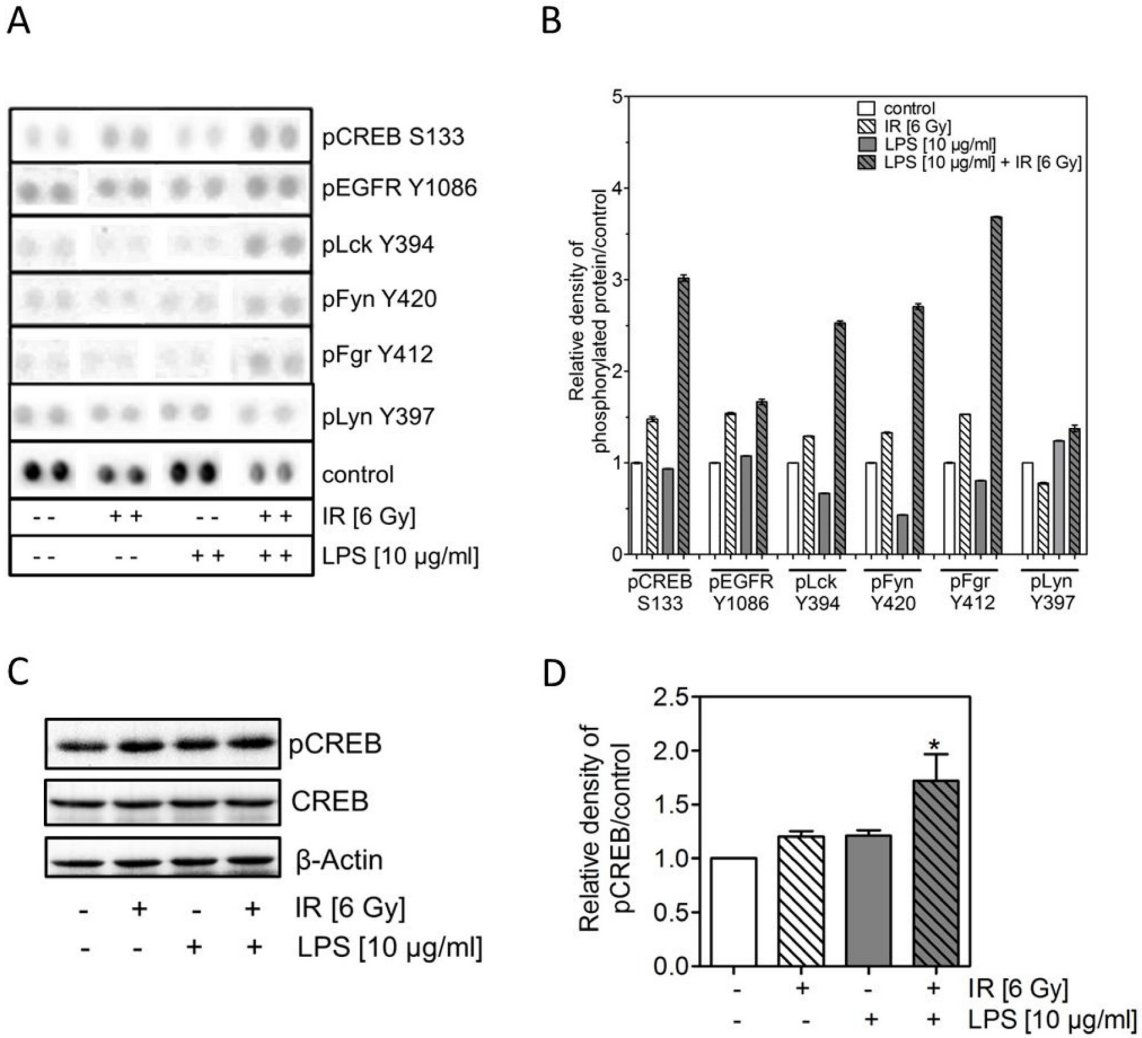
LPS combined with irradiation induces an up-regulation of pCREB (**A**) Representative images of signals phosphorylated forms from a proteome profiling array using the Human Phospho-Kinase Antibody Array Kit of H1975 cells 24 h after treatment with and without 10 μg/ml LPS and irradiation with 0 or 6 Gy. (**B**) Quantitative analyses of results of A) are presented as mean ± SEM. (**C**) Representative western blots of pCREB, CREB in LPS- (10 μg/ml) treated and/or irradiated (6 Gy) H1975 cells after 24 h; ß-actin was used as loading control. (**D**) Densitometric analyses of pCREB bands after normalization to ß-actin of H1975 cells after LPS and/or irradiation, as indicated (±). Western blot data are presented as mean ± SEM, *n* ≥ 3, ^*^p < 0.05 for comparison versus control, IR, LPS as determined by ANOVA following by Bonferroni's Multiple Comparison Test.

To understand the underlying mechanism of the LPS-induced radioresistance further experiments were carried out with H1975 cells. In a first step, H1975 cells were incubated with 10 μg/ml LPS followed by irradiation (6 Gy) as described above and 24 h after treatment a proteome profiling array was performed for 43 multiple human kinases using the Human Phospho-Kinase Antibody Array Kit (Figure 3A). After single treatment with either LPS (10 μg/ml) or irradiation (6 Gy) for many kinases a change in phosphorylation was seen (Supplementary Figure 1A and 1B). However, after the combined treatment a more than additive up-regulation was solely detected for few of them, namely the phosphorylated form of cAMP response element-binding protein (CREB), the lymphocyte-specific protein tyrosine kinase (Lck), the tyrosine-protein kinase Fyn (Fyn) and the tyrosine-protein kinase Fgr (Fgr) (Figure 3A and 3B). For all other kinases the combined treatment did not result in an additive increase but was mostly identical to the effect of irradiation or LPS treatment alone (Figure 3A and 3B, Supplementary Figure 1A and 1B).

Because Lck, Fyn, Fgr are all members of the SRC family and upstream of CREB we verified the enhanced phosphorylation of CREB by western blotting (Figure 3C). In line with the expression data only a moderate increase (1.2) was seen for pCREB phosphorylated at S133 when treated by irradiation or LPS alone (Figure 3D). In contrast, again a significant increase (1.7) was observed for pCREB, when H1975 cells were exposed to the combined treatment.

### Inhibition of CREB binding protein (CBP) decreases the LPS-induced radioresistance

**Figure 4:**
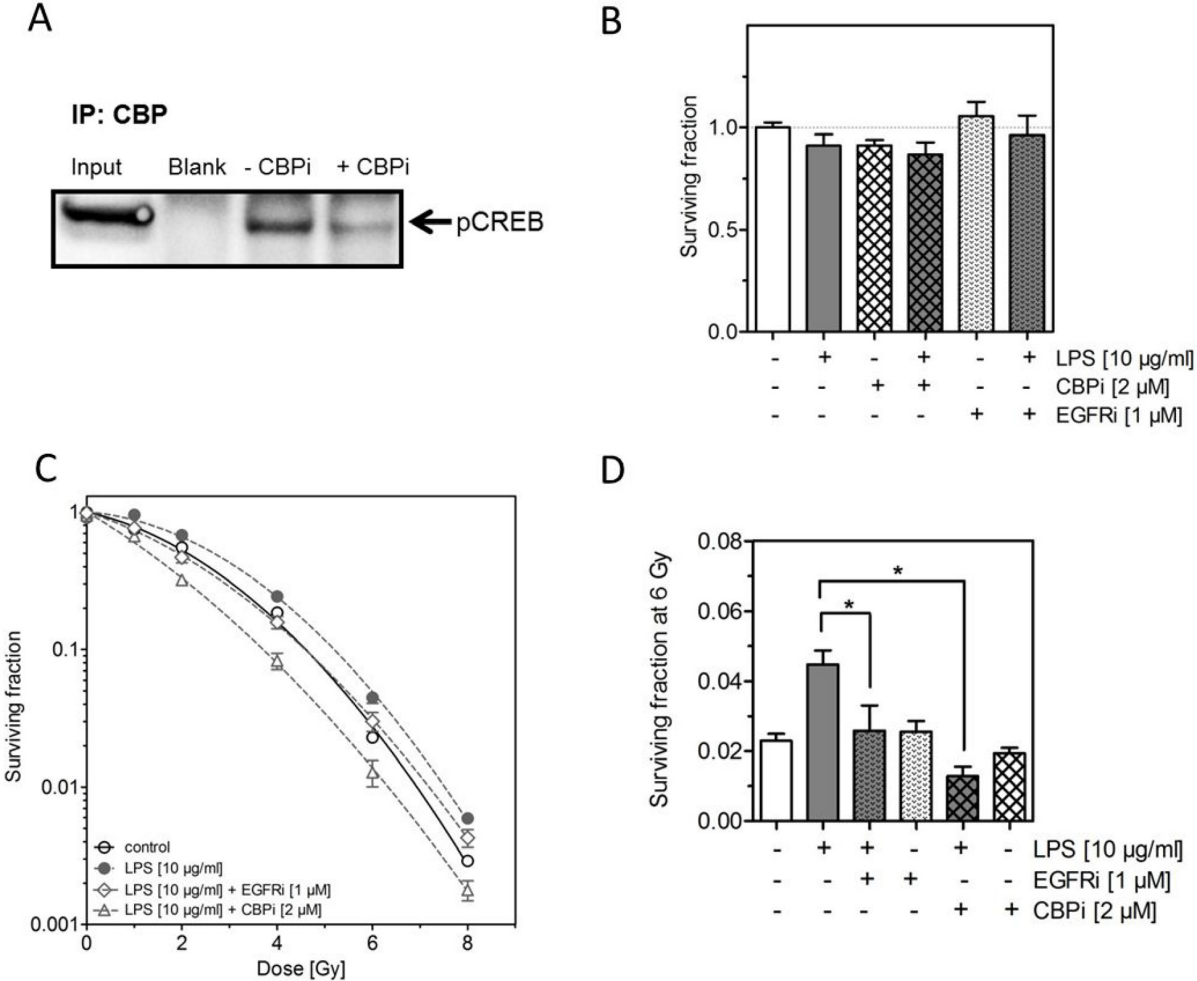
Inhibition of CREB binding protein (CBP) abrogates the LPS-induced radioresistance (**A**) Representative image of IP against CBP followed by western blot of pCREB in H1975 cells 24 h after treatment with LPS (10 μg/ml) and 6 Gy with or without ICG-001 (CBPi) (2 μM), as indicated (±). (**B**) Survival fractions of LPS- (10 μg/ml) or sham-treated (control) H1975 cells with or without CBPi or EGFRi, as indicated (±). (**C**) Survival fractions of LPS- (10 μg/ml) or sham-treated (control) H1975 cells after irradiation with 0–8 Gy with or without CBPi (triangle) or EGFRi (diamond). (**D**) Survival fractions at 6 Gy of LPS- (10 μg/ml) or sham-treated (control) H1975 cells with or without CBPi or EGFRi, as indicated (±). Data are presented as mean ± SEM, *n* ≥ 3, ^*^p < 0.05 for comparison versus LPS at 6 Gy as determined by ANOVA following by Bonferroni's Multiple Comparison Test.

To clarify if the increased phosphorylation of CREB (S133) detected after treatment with LPS and irradiation was causally involved in the LPS-induced radioresistance in H1975, we used the specific CBP inhibitor (CBPi) ICG-001 [29, 30]. We also measured the impact of EGFR using the EGFR kinase inhibitor (EGFRi) AG1478 [31]. The effect of CBPi on the phosphorylation of CREB was successfully confirmed by Co-IP (Figure 4A). Both inhibitors, CBPi and EGFRi, either alone or combined with LPS were found to have no effect on cellular survival (Figure 4B). Interestingly, when CBPi was added to the combined treatment of irradiation and LPS, besides the clear abrogation of the LPS-induced radioresistance also a significant further increase in radiosensitivity was observed (Figure 4C and 4D). In contrast, when EGFRi was added to the combination of LPS and irradiation only an abrogation of the LPS-induced radioresistance was seen (Figure 4C and 4D). Both, CBPi and EGFRi combined with irradiation alone, were found to have no effect on radiosensitivity as shown for an X-ray dose of 6 Gy (Figure 4D). These data demonstrate that pCREB appears to have a prominent role in the LPS-induced radioresistance and that this effect only partially depends on EGFR.

### Inhibition of CBP impairs DNA double-strand break in cells treated by LPS

**Figure 5:**
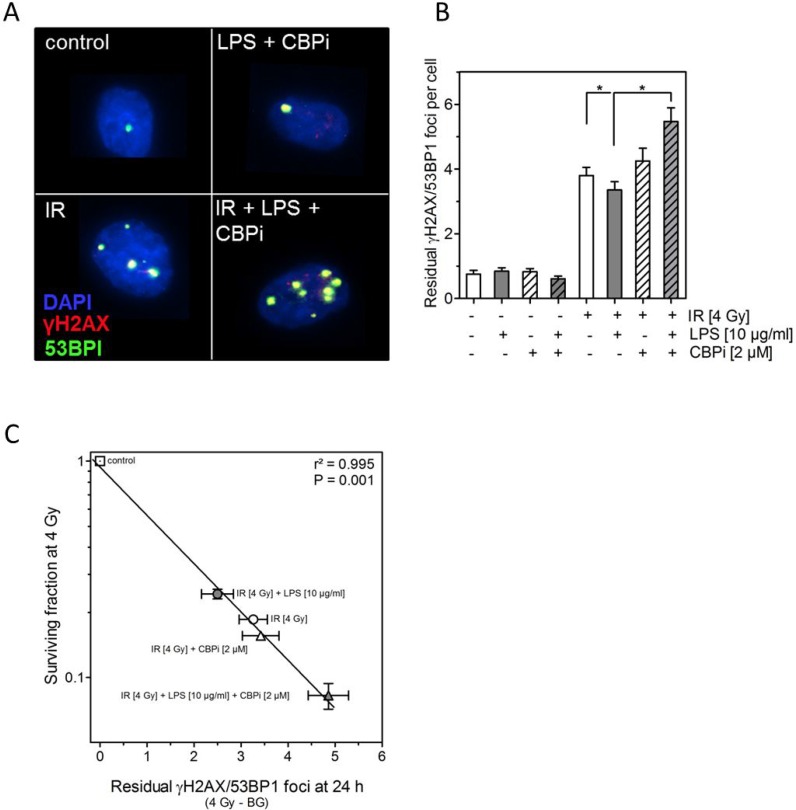
Inhibition of CBP (CBPi) impairs DNA double-strand break in cells treated by LPS (**A**) Representative images of residual γH2AX/BP53 foci in LPS- (10 μg/ml) or sham-treated (control) H1975 cells detected 24 h after irradiation (IR) with 4 Gy and with or without CBPi (2 μM). Magnification, objective 60×. (**B**) Quantification of the number of residual γH2AX/BP53 foci measured 24 h after irradiation (IR) with 4 Gy and with or without CBPi (2 μM) in LPS (10 μg/ml) or sham-treated (control) H1975 cells, as indicated (±). Quantification was performed by counting at least 200 nuclei per sample. Data are presented as mean ± SEM, *n* ≥ 3, ^*^p < 0.05 for comparison versus IR and IR + LPS as determined by ANOVA following by Bonferroni's Multiple Comparison Test. (**C**) Association between the survival fractions at 4 Gy and the number of residual γH2AX/BP53 foci detected 24 h after irradiation with 4 Gy (the number of foci in the corresponding unirradiated cells was subtracted as background) of LPS- (10 μg/ml) or sham-treated (control) and with or without CBPi (2 μM) treated H1975 cells. Data were analyzed by linear regression analysis.

We finally asked whether the changes in radiosensitivity seen here, when irradiation was combined with LPS alone or together with CBP inhibitor, may also result from an altered DSB repair capacity. DSB repair was assessed by counting the number of co-localized γH2AX/53BP1 foci 24 h after irradiation, which is known to be an excellent read-out for an altered DSB repair [32–34] (Figure 5A). For unirradiated cells (control, LPS, CBPi, LPS+CBPi) on average one residual γH2AX/53BP1 foci was counted per nucleus (Figure 5B). Upon treatment with 4 Gy this number increased to about four residual γH2AX/53BP1 foci per nucleus. There was a slight, but statistically significant reduction when irradiation was combined with LPS, while no change was seen when irradiation was combined with the CBPi alone. However, when CBPi was added to the combination of LPS and irradiation a significant increase was seen to about five residual γH2AX/53BP1 foci per nucleus (Figure 5B). This variation in residual γH2AX/53BP1 foci was found to correlate with the differences in surviving fraction seen after the respective treatments (Figure 5C). These data strongly indicate that the modulation of the cellular radiosensitivity caused by LPS alone or in combination with CBPi appear to result from its affected DSB repair.

## DISCUSSION

The purpose of the current study was to investigate the effect of the bacterial pathogenicity factor LPS on the response to radiotherapy in several NSCLC cell lines. The clinical background is given by the observation that pulmonary bacterial infections worsen prognosis of lung cancer patients [5]. However, it remains unclear, if this impairment of prognosis is a simple epiphenomenon of infections, or if bacterial pathogens are causally involved in the development of therapy resistance in NSCLC. In this context, the response to radiotherapy is of particular relevance, as it is the backbone of both curative and palliative therapeutic settings in NSCLC [35, 36].

LPS are the major pathogenic factors of gram-negative bacteria, which are the mostly seen in lung cancer patients [4, 6–8]. LPS are known to activate TLR-4 dependent pathways, which also require the protein kinase activity of the epidermal growth factor receptor (EGFR) [26, 37]. Therefore, three NSCLC cell lines were selected for this study strongly differing in the LPS response with H1975 (EGFR driver mutation) and A549 (EGFR wildtype) both showing a TLR-4 expression and H520 (EGFR-deficient) with a low TLR-4 expression (Table 1).

It was previously shown by us that LPS effectively stimulate tumor growth in various experimental models of NSCLC *in vitro*, *ex vivo* and *in vivo* using the A549 cell line [9]. It is now observed that LPS per se have no effect on the colony forming capacity of H1975, H520 and A549 (Figure 1). These results illustrate a new aspect of LPS to modulate the biology of NSCLC as LPS in fact induce proliferation [9, 38–40] but have no effect on the clonogenicity of tumor cells. Clonogenicity of tumor cells is an important factor for tumor development, progression and recurrence after treatment. It is known that the radiosensitivity of tumor cells *in vitro* well correlates to the tumor´s response to radiotherapy *in vivo* [41]. Thus, the existence of a correlation of stemness *in vivo* and clonogenicity *in vitro* was suggested pointing out the relevance of intrinsic radiosensitivity of cancer stem cells for radiosensitivity of tumors of different histologies [42–45].

Interestingly, we revealed for the first time that LPS are able to induce a significant radioresistance in NSCLC cell lines showing an expression of the LPS binding receptor TLR-4 (Table 1 and Figure 2). This result reveals that the effect of LPS on radiation response also depends on the formation of an active LPS/TLR-4 complex and gives a proof that the observed effect was specifically mediated by LPS. Furthermore, it is known, that in NSCLC a high expression of TLR-4 was found and the level of TLR-4 correlated with the malignancy of these tumors [46]. Thus, the currently shown interaction of LPS with TLR-4 positive NSCLC cells resulting in resistance to radiotherapy may well be operative *in vivo* and may explain the impaired prognosis of TLR-4 positive patients [5].

The LPS/TLR-4 complex is able to stimulate numerous pathways [24]. To investigate which LPS/TLR-4-dependent pathways were responsible for the observed radioresistance we performed a proteome profiling array. It was found that the LPS and irradiation induced a more than additive up-regulation of the cAMP response element-binding protein (CREB) pathway including phospho-Lck, -Fyn, -Fgr, which are all members of the SRC family, finally resulting in the enhanced phosphorylation of CREB, when H1975 cells were treated with both LPS and irradiation (Figure 3). The functional relevance of CREB in mediating LPS-induced radioresistance was proven by the efficacy of CBPi, restoring radiosensitivity in LPS-treated cells.

CREB is a member of the CREB/ATF family of transcription factors that play a key role in the nuclear responses to a variety of external signals that lead to cell growth and proliferation, differentiation, apoptosis and survival [47–49]. CREB is phosphorylated at serine/threonine residues depending upon the stimuli from extracellular components and several upstream kinases [50]. Activated/phosphorylated CREB recruits its transcription co-activator, CREB-binding protein (CBP) to a cAMP response element (CRE) region of target genes [51]. This recruitment of CBP is a critical step for the transcriptional activation of CREB [52]. Several previous studies showed that CREB is highly upregulated and hyperphosphorylated in non-small cell lung cancer (NSCLC) tumor specimen and that this upregulation is significantly associated with poor survival rates [53–55]. Against this background, the currently observed *in vitro*-observations may be of major relevance *in vivo* in NSCLC. Besides these facts, there are already several reports, which showed that LPS induce pCREB for example in human monocytes, normal lung tissue, and in the A549 cell line [24, 56–58], indicating a close interaction between LPS and the CREB pathway. Our results revealed that LPS per se induce the phosphorylation of CREB in H1975 cells about the factor 1.2 (Figure 3D).

After irradiation a similar increase of phosphorylated CREB about the factor 1.2 was measured (Figure 3D). In line with our results there are several reports, which showed that irradiation activate the CREB pathway in different types of cancer (hematological, gastrointestinal, lung, prostate) [49]. These results may be explained by the fact that pCREB is already highly upregulated in this cell line like Aggarwal *et al*. assumed [55] and therefore LPS *per se* and irradiation *per se* produced only a slight increase the phosphorylation of CREB in H1975 cells.

However, the phosphorylation levels of CREB were significantly increased upon combined treatment with LPS and irradiation (Figure 3). The relevance of this considerably enhanced level of pCREB for the LPS-induced radioresistance was demonstrated by using the specific inhibitor ICG-001 of the CREB-binding protein (CBP) [29, 30]. Interestingly, inhibition of CBP not only resulted in a complete abrogation of the LPS-induced radioresistance but even caused to a further reduction in radiosensitivity (Figure 4). The specificity of the currently used inhibitor ICG-001 was proven by Co-Immunoprecipitation, clearly showing a strong reduction in the phosphorylation level of CREB when ICG-001 (CBPi) is present (Figure 4A).

It was demonstrated that the changes in cell survival are well correlated with the respective differences in the numbers of residual DNA-DSBs, as recorded after the identical treatments using the γH2AX/53BP1 foci technique (Figure 5). These data demonstrate that LPS modulate cellular radiosensitivity via the CREB pathway due its effect on DSB repair capacity.

There are already several reports indicating that CREB is involved in DSB repair [49]. It was shown for CHO cells that a down-regulation of CREB in a dominant-negative mutant results in a depressed DSB repair, which will then also lead to an enhanced cellular radiosensitivity [59]. The reduction in DSB repair was shown to result from a down-regulated non-homologous End-joining (NHEJ) [60], one of the two major DSB repair pathways acting in mammalian cells, because inhibition of CREB was found to result in an impaired acetylation of Ku70, which is a key component of NHEJ [61]. Overall, our current data suggest that the radioresistance observed upon LPS treatment, might be due to an increased NHEJ activity caused by the elevated amount of phosphorylated CREB induced by the combined treatment (Figure 3). And, vice versa, the abrogation of this radioresistance with even a radiosensitization when CBPi is added to this combination is considered to result from a strong reduction of NHEJ down to a level, which is below that of untreated cells.

In contrast to pCREB no additive increase in pEGFR was seen, when LPS was combined with irradiation (Figure 3A and 3B). Accordingly, when EGFR was inhibited during the combined treatment with LPS and irradiation a clear abrogation of the LPS-induced radioresistance was achieved, but with no further radiosensitization below the irradiated control as observed under CBPi (Figure 4). These findings are in line with the concept that EGFR is needed, but not directly involved in the LPS-TLR-4 pathway, as described by De *et al*. [26]. They have shown that the LPS induced pathway via TLR-4 strongly depends on a crosstalk to EGFR by pLyn. Maybe another member of the SRC family like Lck, Fyn, Fgr are in particular after irradiation mediating in the crosstalk of EGFR and TLR-4, because pLyn was not increased after LPS and irradiation (Figure 3A and 3B). Further analyses are necessary to prove this proposed mechanism in detail.

The observation made here, that LPS are able to induce radioresistance in TLR-4 positive NSCLC cell lines, which, however, can be abrogated by the inhibition of CBP with even a radiosensitization, is maybe of interesting clinical relevance. NSCLC is often associated with pulmonary infection, where circulating LPS are present [4]. Targeting pCREB in combination with radiotherapy may be a novel option for a more effective and specific therapy of these cancer patients, who are, so far, characterized by an extremely poor outcome.

In conclusion, it is shown here that LPS have no impact on the clonogenicity of tumor cells, but induce a radioresistance in TLR-4 and EGFR expressing NSCLC cells. However, this resistance can be abrogated by the inhibition of CBP, which even results in a radiosensitization. These data indicate that the combination of CBPi and radiotherapy might be a promising new strategy to improve the outcome of NSCLC patients, which suffer from pulmonary infections.

## MATERIALS AND METHODS

### Cell culture

Experiments were performed with the human NSCLC adenocarcinoma cell lines H1975 (CRL-5908) which has a L858R/T790M double mutations in EGFR, A549 (CCL-185) which are EGFR wild type and the human squamous cell carcinoma cell line H520 (HTB-182) which is EGFR-negative obtained from the American Type Culture Collection (ATCC; Manassas, VA, USA). Cells were maintained in RPMI 1640 (E15-840; PAA Laboratories GmbH, Pasching, Austria) supplemented with 10% fetal bovine calf serum (FBS, Sigma-Aldrich, St. Louis, MO, USA) and 105 U/l penicillin, and 100 mg/l streptomycin (Pan - Biotech GmbH, Aidenbach, Germany), at 37°C in humidified atmosphere containing 5% CO_2_ in air. Authentication of all used cell lines was performed by short tandem repeat analysis at the German Collection of Microorganisms and Cell Cultures (DSMZ, Germany).

### Treatment with LPS

For the LPS treatment the cells were seeded on cell culture flasks (Falcon, Corning, Inc., Corning, NY, USA) and grown for 24 h. The cells were stimulated with 0.1, 1 or 10 μg/ml highly purified LPS 0111:B4 from *E. coli* (Sigma-Aldrich, St. Louis, MO, USA) for 16 h before irradiation and after irradiation during the experimental setup with sham-treated cells used as control. Trypan blue (Sigma-Aldrich, St. Louis, MO, USA) staining was used for obviate any toxicity of used LPS doses in our experimental setups.

### Treatment with the CBP-inhibitor ICG-001 and the EGFR-inhibitor AG1478

The CBP-inhibitor ICG-001 (Merck Millipore, Darmstadt, Germany) and the EGFR-inhibitor AG1478 (Merck Millipore, Darmstadt, Germany) were prepared and diluted according to manufacturer instructions. Cells were incubated with 2 μM ICG-001 or 1 μM AG1478 1 h before irradiation and after irradiation during the experimental setup. The control group was treated with corresponding DMSO (Dimethylsulfoxid, Sigma-Aldrich, St. Louis, MO, USA) dilution.

### X-irradiation

For X-irradiation, a 6-MeV X-ray beam generated by a clinical linear accelerator was used. The maximum dose rate was 4 Gy/min. X-irradiation was delivered at room temperature and applied doses ranged from 0 to 8 Gy. Cell culture flasks were arranged between 15 mm water-equivalent plates to generate doses maximum in the cell layer.

### Colony formation assay

For colony formation assay cells were seeded in 6 cm culture dishes after irradiation (0–8 Gy), the cell number was determining with respect to the plating efficiency and dose in order to obtain 100 colonies. After incubation for 10–14 days, the cells were fixed, stained with 0.1% crystal violet (Sigma-Aldrich, St. Louis, MO, USA) and colonies >50 cells were counted. Surviving fractions (SF) were calculated as published previously [62, 63].

### Proteome profiling

Proteome profiling arrays were performed using Human Phospho-Kinase Antibody Array Kit (#ARY003B, R&D Systems, Minneapolis, MN, USA) according to manufacturer instructions. Cells were grown in T25 culture flasks, rinsed with PBS, and lysed. Protein concentration was estimated with BCA Protein Assay Reagent (Thermo Fisher Scientific Inc., Waltham, MA, USA) and 2000 μg of each cell lysate was added to pre-blocked antibody array membranes for incubation. Membranes were treated with detection antibody cocktail followed by streptavidin-HRP, and signal was detected using the chemiluminescence method as instructed. The Array was analyzed by BioDocAnalyze Software (Analytik Jena AG, Jena, Germany). The relative expression levels are shown normalized to the control group (*n* = 2).

### Western blot

Cells were rinsed with PBS prior to adding buffer 6 (R&D Systems, Minneapolis, MN, USA) for protein isolation. Protein concentration was determined with the BCA assay (Pierce-ThermoFisher Scientific, Rockford, IL, USA). After SDS-PAGE and transfer of proteins onto a polyvinyl difluoride (PVDF) membrane; nonspecific sites were saturated with 5% milk. Incubation was performed overnight (4°C) with the following primary antibodies: anti-phospho-CREB phospho S311 (ab32096, diluted 1:500, Abcam, Cambridge, UK), anti-CREB (ab32515, diluted 1:500, Abcam, Cambridge, UK), and anti-beta-Actin rabbit mAb (#4970, diluted 1:2000, Cell Signaling Technology, Cambridge, UK). Immunodetection was performed by incubation 1 h with peroxidase-conjugated secondary antibody goat anti-rabbit IgG (#7074, diluted 1:2000, Cell Signaling Technology, Cambridge, UK), with an ECL system (Thermo Fisher Scientific Inc., Waltham, MA, USA). The signals were quantified by densitometric scanning (Bio Rad ChemiDoc XRS+, Bio-Rad Laboratories, Inc., Hercules, USA).

### Co-Immunoprecipitation

For the Co-Immunoprecipitation the cells were washed with PBS. The cells were lysed by incubating 20 min with Co-IP buffer (50 mM HEPES, 150 mM NaCl, 1 mM EGTA, 10% Glycerol, 1% Triton-100). After pre-cleaning an equal amount of proteins (>500 μg) were incubated with an antibody against Anti-KAT3A/CBP antibody (ab2832, 10 μl, Abcam, Cambridge, UK) or Rabbit IgG, polyclonal (ab27478, 10 μl, Abcam, Cambridge, UK) for the isotype control over night. After the incubation with this antibody 50 μl protein A/G Plus-agarose beads (Santa Cruz Biotechnology, Dallas, TX, USA) were added and incubated overnight a 4°C. After, the beads were washed 5 times with Co-IP buffer. The samples were resuspended in 2× Laemmli buffer with 10% beta-Mercaptoethanol and 20 mM DTT and boiled for 10 min. Proteins were analyzed by SDS-PAGE and Western Blot. For the Western Blot the incubation was performed overnight (4°C) with the primary antibody anti-phospho-CREB (phospho S311) (ab32096, diluted 1:500, Abcam, Cambridge, UK). Immunodetection was performed by incubation 1 h with peroxidase-conjugated secondary antibody goat anti-rabbit IgG (#7074, diluted 1:2000, Cell Signaling Technology, Cambridge, UK), with an ECL system (Thermo Fisher Scientific Inc., Waltham, MA, USA).

### Immunofluorescent microscopy and quantification of γH2AX/53BP-1 foci

Cells were grown on glass cover slips for 24 h. After incubation with LPS for 16 h cells were irradiated with a dose of 4 Gy. For analysis of γH2AX/53BP1 foci, cells were fixed and stained 24 h after irradiation with 4% paraformaldehyd (Carl Roth GmbH + Co. KG, Karlsruhe, Germany)/PBS (Sigma-Aldrich, St. Louis, MO, USA) for 10 min and stored at 4°C. Fixed cells were permeabilized with 0.2% Triton X-100 (SERVA Electrophoresis GmbH, Heidelberg, Germany), 1% BSA for 10 min and blocked in 3% BSA for 1 h. Primary antibody incubation was done for 1 h at room temperature using the following antibodies: Anti-phospho-Histone γH2A.X (Ser139) close JBW301/mouse (1:500, Merck Millipore, Billerica, MA, USA), 53BP1 anti-rabbit (1:500, Bio-Techne-Novus Biologicals Minneapolis, MN, USA). After washing three times with 0.5% Tween 20/PBS for 10 min, the cells were incubated for 1 h with secondary anti-mouse Alexa-fluor 594 (1:800, Invitrogen, Carlsbad, CA, USA) and anti-rabbit Alexa-fluor 488 (1:1200, Invitrogen, Carlsbad, CA, USA). Cells were again washed three times and mounted in ProLong Gold antifade reagent (Invitrogen, Carlsbad, CA, USA) including DAPI for staining of nuclei. Immunofluorescence was analyzed using the IX81 microscope (objective: 60x, Olympus, Shinjuku, Tokio, Präfektur Tokio, Japan) and Xcellence Software (Olympus¸ Shinjuku, Tokio, Präfektur Tokio, Japan). For analysis z-stacked images were taken from each sample and foci counted manually. The number of foci in irradiated samples was calculated by background subtraction from non-irradiated controls. These experiments were performed at least two times in duplicates and at least 200 nuclei were counted.

### RNA isolation and real-time RT-PCR

For quantification of *TLR-4* and *EGFR* mRNA, total RNA was extracted with RNeasy Mini Kit (QIAGEN N.V., Hilden, Germany) according to the manufacturer's protocols. Extracted RNA was quantified with Nano Drop (PeqLab, Erlangen, Germany). The cDNA was synthesized by RT (Bio-Rad, München, Germany). Real-time PCR was performed using 1 μg of cDNA, SYBR Green PCR Master Mix (Bio-Rad, München, Germany) and 0.05 M forward/reverse primers; specific primers used for sequence detection were as follows:

TLR-4: 5′ccagcattccaatttgaaacaaatg3′ (forward) and 5′gagaggtccaggaaggtcaagtttc3′ (reverse).

EGFR: 5´gtgaaaacaccgcagcatgt3′ (forward) and 5′cccgtagctccagacatcac3′ (reverse)

PBGD: 5′cagcttgctcgcatacagac3′ (forward) and 5′gaatcttgtcccctgtggtg3′ (reverse).

Real-time-reactions were performed as described before [64]. The mRNA expressions were expressed as –ΔCt value (Ct value gene of interest – Ct value gene of reference gene [PBGD]).

### Calculation and statistical analyses

If not otherwise indicated, results are presented as mean values ± standard errors of the mean (SEM) for at least 3 independent experiments. The level of significance was evaluated by one-way ANOVA, followed by Bonferroni's Multiple Comparison Test. Differences at *p-*values of < 0.05 were considered statistically significant and are indicated in the figures by an asterisk.

## SUPPLEMENTARY MATERIALS FIGURE


