# Supplementary material for: Targeting CREB-binding protein overrides LPS induced radioresistance in non-small cell lung cancer cell lines
Source: Oncotarget. 2018 Jun 22;9(48):28976–88. doi: 10.18632/oncotarget.25665 (PMC6034744; doi:10.18632/oncotarget.25665)
Supplement: Supplementary file 1 [file oncotarget-09-28976-s001.pdf]

Targeting CREB-binding protein overrides LPS induced radio-resistance in non-small cell lung cancer cell lines

SUPPLEMENTARY MATERIALS

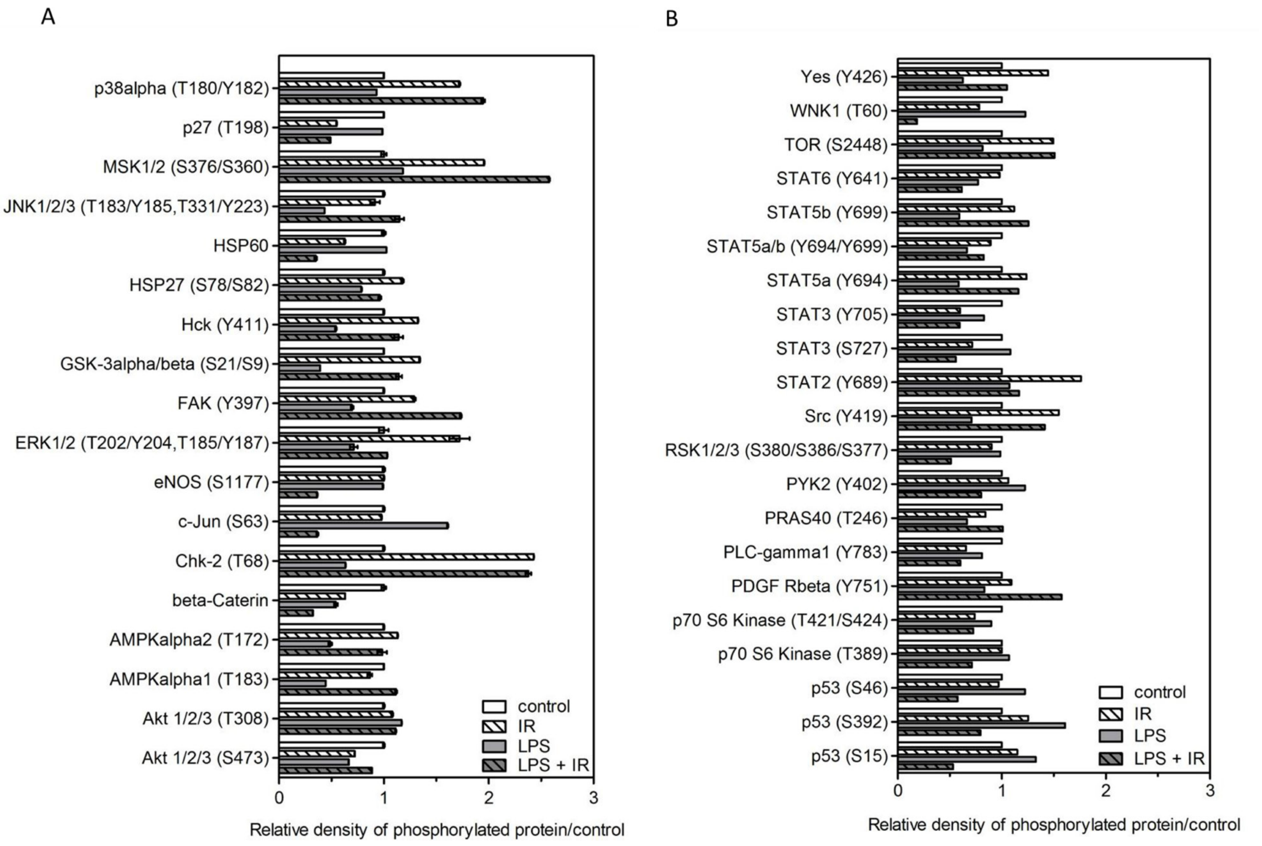

Supplementary Figure 1: Quantitative analyses of phosphorylated forms from the remaining results of the proteome profiling array using the Human Phospho-Kinase Antibody Array Kit of H1975 cells 24 h after treatment with and without 10 µg/ml LPS and irradiation with 0 or 6 Gy. (**A**) Phosphorylated forms in alphabetical order beginning by A to P. (**B**) Phosphorylated forms in alphabetical order beginning by P to Y. Data are presented as mean ± SEM.
